# Self-Thickening Materials Derived from Phenylpropanoid Ene Reactions

**DOI:** 10.3390/molecules30050977

**Published:** 2025-02-20

**Authors:** Atanu Biswas, Huai N. Cheng, Bret Chisholm, Ryan Beni, Zengshe Liu, Karl Vermillion, Michael Appell, Kelton Forson, Omar El Seoud, Carlucio R. Alves, Roselayne F. Furtado

**Affiliations:** 1 National Center for Agricultural Utilization Research, USDA Agricultural Research Services, 1815 N. University Street, Peoria, IL 61604, USA; 2 Southern Regional Research Center, USDA Agricultural Research Service, 1100 Allen Toussaint Boulevard, New Orleans, LA 70124, USA; 3 Department of Chemistry, Tennessee State University, Nashville, TN 37209, USA; 4 Institute of Chemistry, University of São Paulo, São Paulo 05508-000, SP, Brazil; 5 Chemistry Department, State University of Ceará, Silas Munguba Av. 1.700, Fortaleza 60740-020, CE, Brazil; 6 Embrapa Agroindústria Tropical, Rua Dra. Sara Mesquita 2270, Fortaleza 60511-110, CE, Brazil

**Keywords:** allylbenzene, diethyl azodicarboxylate, ene reaction, eugenol, methyl eugenol, phenylpropanoids, self-thickening

## Abstract

In this work, we report the observation of uncatalyzed ene reactions between several phenylpropanoid compounds and diethyl azodicarboxylate (DEAD). For allylbenzene, the reaction produces the ene product at molar ratios of up to 1:2 of allylbenzene to DEAD. At higher levels of DEAD, more complex reactions are observed. For the reaction between methyl eugenol and DEAD, similar ene reaction products have been found. However, the reaction of eugenol with DEAD is more complex; in addition to the ene reaction, other reactions happen at the same time. Most of the structures of the resulting products have been elucidated using NMR spectroscopy (^1^H, ^13^C, and 2D), and the findings have been further corroborated by FTIR analysis. Interestingly, these products appear to undergo molecular aggregation, which results in self-thickening in their neat form. However, the viscosity significantly decreases upon dilution with a solvent. This self-thickening property suggests their potential use as thickening agents in organic solvent formulations.

## 1. Introduction

Alder’s ene reactions involve the interaction of an alkene (ene) with a compound containing an electron-deficient multiple bond (enophile) to form a new sigma bond, a process that often occurs with high regio- and stereoselectivity [1,2,3]. The relevance of Alder’s ene reactions lies in their versatility and efficiency in designing complex molecular architectures, making them useful tools in the syntheses of natural products, pharmaceuticals, and advanced materials. Their ability to facilitate the formation of carbon–carbon and carbon–heteroatom bonds under relatively mild conditions further underscores their utility in green chemistry, promoting sustainable practices by minimizing both the energy consumption and the generation of hazardous by-products. Consequently, many ene reactions have been carried out on organic compounds [4,5,6,7]. There have also been some efforts to use this reaction for biological systems [8].

**Scheme 1:**
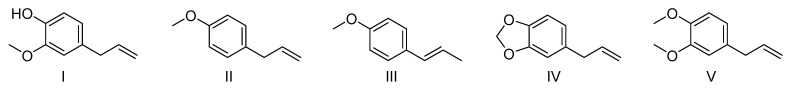
Natural products with allylbenzene functionality: eugenol (I), estragole (II), anethole (III), safrole (IV), and methyl eugenol (V).

Because of current interest in sustainability and green chemistry [9,10,11,12,13], we have been working with agro-based raw materials for research and product development. A molecule of particular interest is allylbenzene, a natural product found in plants like *Alpinia officinarum* and *Daucus carota* [14]. In plant biochemistry, the allylbenzene skeleton is the parent structure for several phenylpropanoids, which are found throughout the plant kingdom [15,16]. For example, this structure is found in various natural products and biobased materials, including eugenol (structure I), estragole (structure II), anethole (structure III), safrole (structure IV), and methyl eugenol (structure V) (Scheme 1).

Eugenol, found in clove oil, nutmeg, cinnamon, and basil, is widely used in flavoring, perfumery, and medicine due to its antimicrobial and analgesic properties [17,18,19]. Estragole, present in tarragon and basil, is commonly utilized in the flavoring industry and holds potential therapeutic value, though it is also being studied for its possible toxic effects [20,21]. Anethole, the primary component of anise, fennel, and star anise essential oils, is a popular flavoring agent known for its antifungal, antibacterial, and anti-inflammatory properties [22,23]. Safrole, found in sassafras oil and spices like nutmeg, has historical uses in fragrances and flavorings but is now regulated due to its carcinogenic properties [24]. Methyl eugenol occurs naturally in *Pinus densiflora*, *Portenschlagiella ramosissima*, and *Osmorhiza aristata* [21,25]. Together, these compounds highlight the wide-ranging applications and significance of allylbenzene derivatives across different fields, such as culinary arts, perfumery, pharmaceuticals, and biotechnology.

**Scheme 2:**
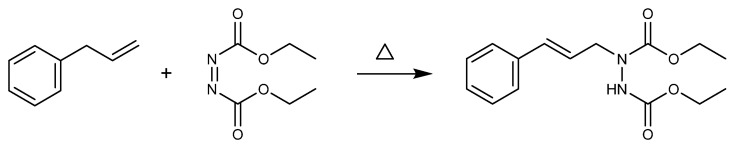
Reaction using heat between allylbenzene and DEAD.

We have previously used ene reactions to study the stereochemistry of olefin–maleic anhydride reactions [4], the reaction of triglycerides with diethyl azodicarboxylate (DEAD) [5] and 4-phenyl-1,2,4-triazoline-3,5-dione (PTAD) [6], and the reaction of cardanol with DEAD [7]. The ene reaction between allylbenzene and DEAD was reported previously [26] (Scheme 2). In this work, we have taken a closer look at the ene reactions involving several phenylpropanoid molecules, i.e., allylbenzene, methyl eugenol, and eugenol, and produced useful and practical molecules. Interestingly, the DEAD derivatives of these molecules exhibit a self-aggregation phenomenon, causing significant increases in viscosity when concentrated but much reduced viscosity when diluted with a solvent.

## 2. Results and Discussion

### 2.1. Allylbenzene Reaction and NMR Analysis

**Figure 1:**
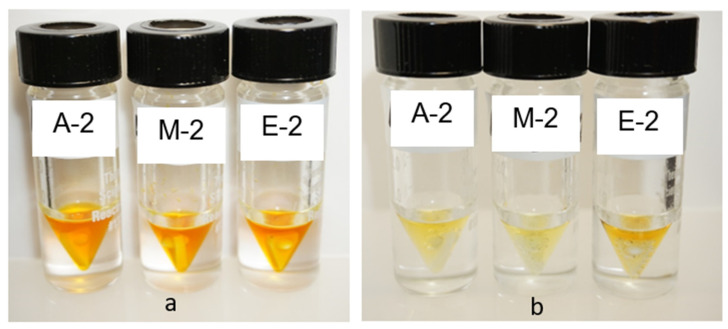
Photographs of (**a**) samples A-2, M-2, and E-2 (at ca. 1:2 molar ratio of alkene/DEAD); (**b**) the same three samples after overnight heating at 90 °C.

The reactions between DEAD and alkenes at several molar ratios of alkenes to DEAD are shown in Table 1. Before the reaction, DEAD was a reddish liquid, whereas the three alkenes were colorless. When mixed at a 1:2 alkene/DEAD molar ratio, an orange liquid was observed initially (Figure 1a). After heating overnight at 90 °C, the orange color partly faded, with sample M-2 showing the least color and sample E-2 the most color (Figure 1b).

**Figure 2:**
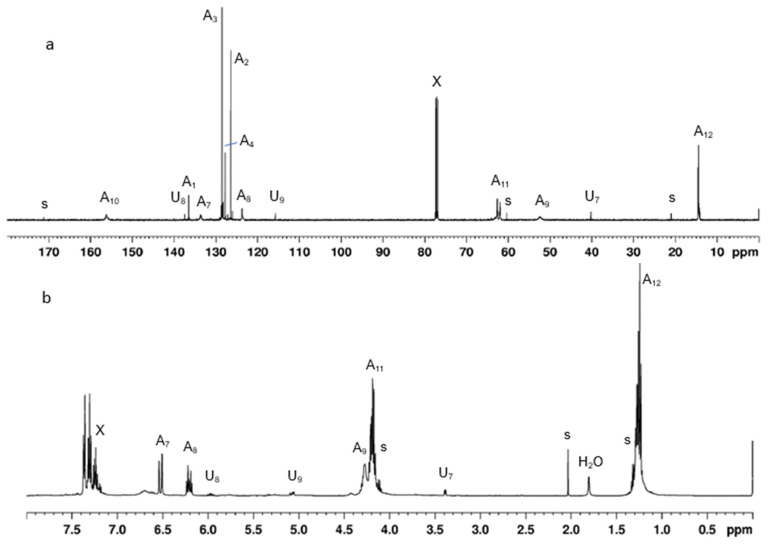
NMR spectra of the 1:2 reaction products between allylbenzene and DEAD (sample A-2): (**a**) ^13^C spectrum and (**b**) ^1^H spectrum. U and A denote unreacted and ene products, respectively; the subscripts correspond to the numbering shown in Scheme 3. The letter X indicates the CDCl_3_ peaks, and the letter s denotes ethyl acetate peaks.

The ^13^C and ^1^H NMR spectra of the reaction product of allylbenzene (AB) and DEAD with the AB:DEAD molar ratio of 1:2.17 (Sample A-2), purified through chromatography, are shown in Figure 2a,b, respectively. The NMR spectral assignments were made by using predicted shifts obtained from the NMRShiftDB computer database [27], ^13^C chemical shift additivity rules [28], and the relevant literature [29,30]. The 2D HSQC and COSY plots for this sample are given in the Supplementary Materials.

**Scheme 3:**
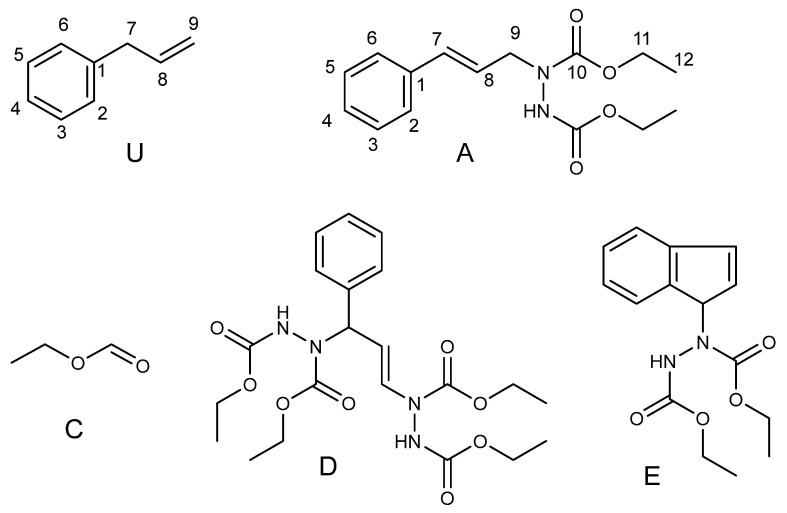
Structures for unreacted allylbenzene (U) and the AB-DEAD ene reaction derivative (A) and three other structures (C,D,E). Structures (U) and (A) are numbered to facilitate NMR assignments.

The spectra in Figure 2 are consistent with the ene product formation between DEAD and AB (structure A) together with some unreacted AB (structure U) (Scheme 3). The ^13^C chemical shifts of the AB-DEAD reaction product are summarized in Table 2. Compared to the starting allylbenzene (structure U), the peaks for the aromatic carbons shift somewhat in structure A because the olefin is now conjugated. The ^13^C shifts for carbons 7, 8, and 9 (structure A) are found at 133.6, 123.8, and 52.6 ppm, respectively, corresponding to the olefin shift due to the ene reaction. The peaks for carboxyl and ethyl functionalities in structure A are found at 156.4, 63.8, and 14.1 ppm. The peak assignments have been directly marked in the ^1^H and ^13^C spectra (Figure 2a,b). Note that in Figure 2a, all the peaks have been assigned to the expected ene structure (structure A) and unreacted AB (structure U); the only other peaks in the spectra are due to the solvents present (CDCl_3_, H_2_O, and ethyl acetate). There are no observable impurities in the sample because there are no other unassigned peaks in Figure 2a. A sample further purified via a silica column was prepared; its NMR spectra are shown in Figure S2 (Supplementary Materials). The elemental analysis showed C 61.2%, H 6.8%, N 9.6%, and O 22.4%; the expected results for structure A are C 61.6%, H 6.8%, N 9.6%, and O 21.9%.

**Figure 3:**
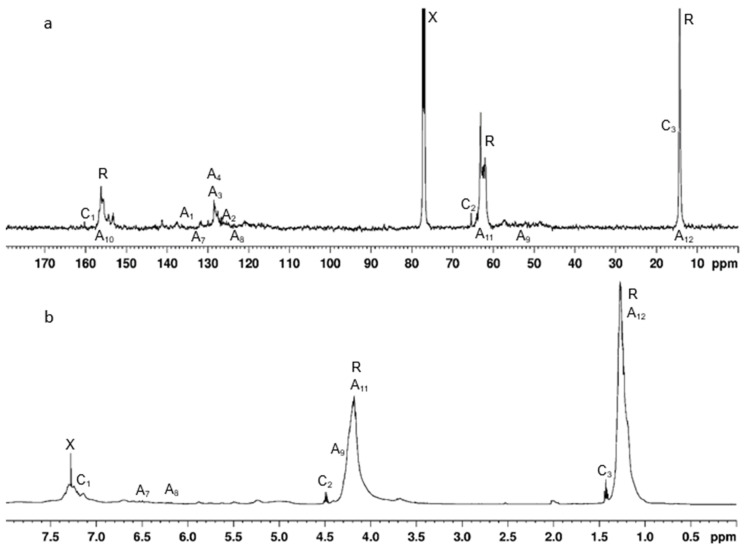
NMR spectra of the 1:3 reaction products between AB and DEAD (sample A-5): (**a**) ^13^C spectrum, (**b**) ^1^H spectrum. A and C denote ene products and ethoxyformaldehyde, respectively; the subscripts correspond to the numbering shown in Scheme 3. R denotes the peaks from residual DEAD moieties after the reaction and X the CDCl_3_ peaks.

At higher AB:DEAD ratios (e.g., sample A-5), the NMR spectra (Figure 3a,b) showed the degradation of surplus DEAD to ethoxyformaldehyde (structure C), a large reduction in the intensities of the AB-DEAD ene product (structure A), and the broadening of all spectral peaks in general. Some ene reaction possibly occurred between DEAD and product A, giving product D. Because of additional small peaks and significant line broadening, other reaction products (e.g., imidazole structure, E) might have also occurred, together with some possible polymerizations [31]. In view of the complexity of the reactions at high DEAD levels, these reactions are not likely to produce useful and practical molecules; thus, we do not recommend the use of higher DEAD levels for the ene reaction, and we have not studied these materials further in this work

Thus, the allylbenzene/DEAD reaction produces the ene product (A) at molar ratios of up to 1:2 of allylbenzene to DEAD, caused by the addition of DEAD to position 9 in allylbenzene, with the olefin shift to the 7 and 8 positions.

### 2.2. Methyl Eugenol and Eugenol Reactions and NMR Analysis

**Scheme 4:**
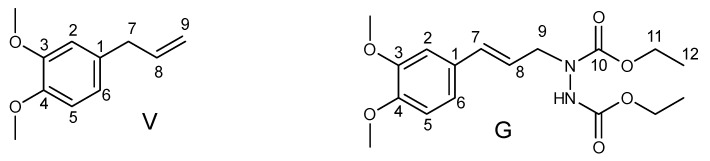
Structures for unreacted methyl eugenol (V) and the methyl eugenol/DEAD ene reaction derivative (G), numbered to facilitate NMR assignments.

**Figure 4:**
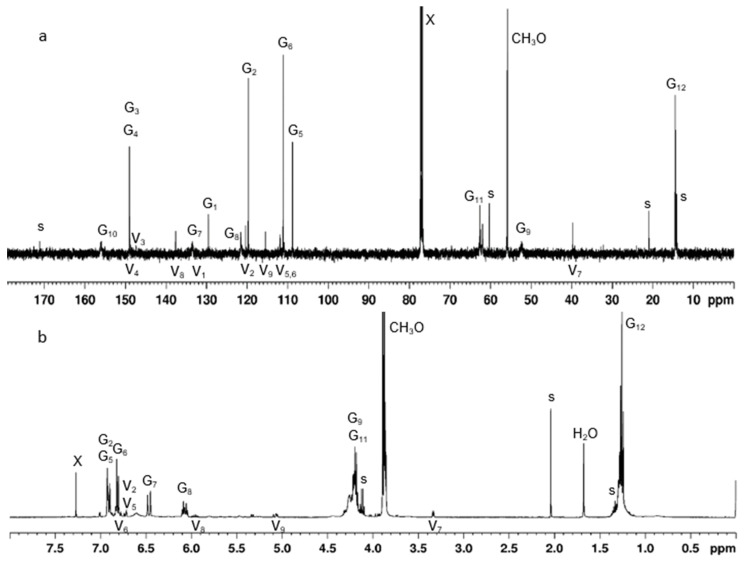
NMR spectra of the reaction products between methyl eugenol and DEAD (sample M-1): (**a**) ^13^C spectrum and (**b**) ^1^H spectrum. V and G denote unreacted methyl eugenol and ene products; the subscripts correspond to the numbering shown in Scheme 4. X denotes the CDCl_3_ peak, and s denotes ethyl acetate peaks.

Methyl eugenol (also known as 4-allyl-1,2-dimethoxybenzene or eugenol methyl ether) (ME, structure V) was also reacted with DEAD, using the same procedure as for allylbenzene. For the ene product (sample M-1) with a 1:1.15 ME:DEAD molar ratio, column chromatography provided a purified compound for analysis. The ^13^C and ^1^H NMR spectra of this ME-DEAD ene derivative are shown in Figure 4a,b, respectively. Again, through the aforementioned assignment strategy, all the main peaks have been assigned. The ^13^C NMR assignments are summarized in Table 2. The appropriate peaks in Figure 4a,b are also labeled with the assignments. The main product is shown to be the ene product (structure G, Scheme 4); note the peaks at 133.5, 121.6, and 52.4 ppm for positions 7, 8, and 9 in Figure 4a. In Table 2, the chemical shifts for the aromatic carbons are slightly different for structures V and G because of the ene reaction. Structure G also has additional peaks at 160.0, 62.3, and 14.4 ppm due to the carboxyl and the ethyl moieties. There is also the presence of some unreacted ME (structure V) in the spectrum. (The 2D COSY and HSQC NMR plots for sample M-1 are given in the Supplementary Materials).

Note that in the ^13^C NMR spectrum of the ME-DEAD product (Figure 4a), all the peaks have been assigned to the expected ene structure (structure G) and unreacted ME (structure V); some solvents (CDCl_3_ and ethyl acetate) are also present. There are no impurities because there are no unassigned peaks in Figure 4a. In addition, an elemental analysis was performed on the sample, showing C 58.1%, H 6.3%, N 7.4%, and O 27.3%; the expected results for structure G are C 57.9%, H 6.8%, N 7.9%, and O 27.2%.

**Scheme 5:**
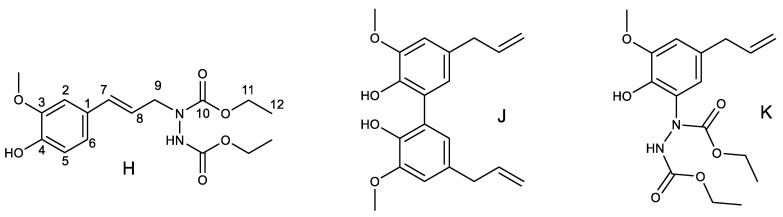
Structures for the eugenol/DEAD ene derivative (H), numbered to facilitate NMR assignments, and two other possible structures produced in the eugenol/DEAD reaction.

**Figure 5:**
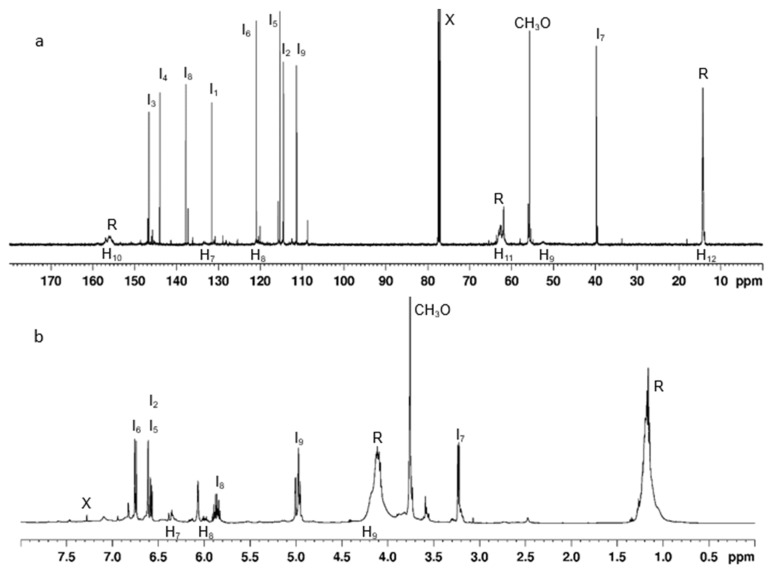
NMR spectra of the reaction products between eugenol and DEAD (sample E-1). (**a**) ^13^C spectrum, (**b**) ^1^H spectrum. I and H denote unreacted eugenol and ene products, respectively; the subscripts correspond to the numbering shown in Scheme 5. R denotes the peaks from residual DEAD moieties after the reaction, and X represents the CDCl_3_ peaks.

The same reaction between eugenol (structure I) and DEAD was also studied via the same procedure. However, upon analysis, the reaction was found to be more complex because the hydroxy group in eugenol affected the outcome of the reactions. As a result, the NMR spectra (for product E-1, Figure 5a,b) were very complex. Nevertheless, the ^1^H and ^13^C NMR spectra clearly show the presence of the ene reaction product H (Scheme 5), as evidenced by the small peaks at 53 ppm, 120 ppm, and 133.5 ppm (^13^C) and 4.1, 5.85, and 6.38 ppm (^1^H), corresponding to the nuclei at positions 9, 8, and 7, respectively. The estimated ^13^C shifts are shown in Table 2. In addition to structure H, there are also other peaks that appear in the ^13^C and ^1^H spectra. Some of them may perhaps be due to the biphenyl structure (J), the phenylhydrazine structure (K) (Scheme 5), diphenoquinone, and possible mixed reaction products. Structures J and K and diphenoquinone have been previously reported for the reactions between phenol and DEAD [32].

### 2.3. FTIR Analysis

**Figure 6:**
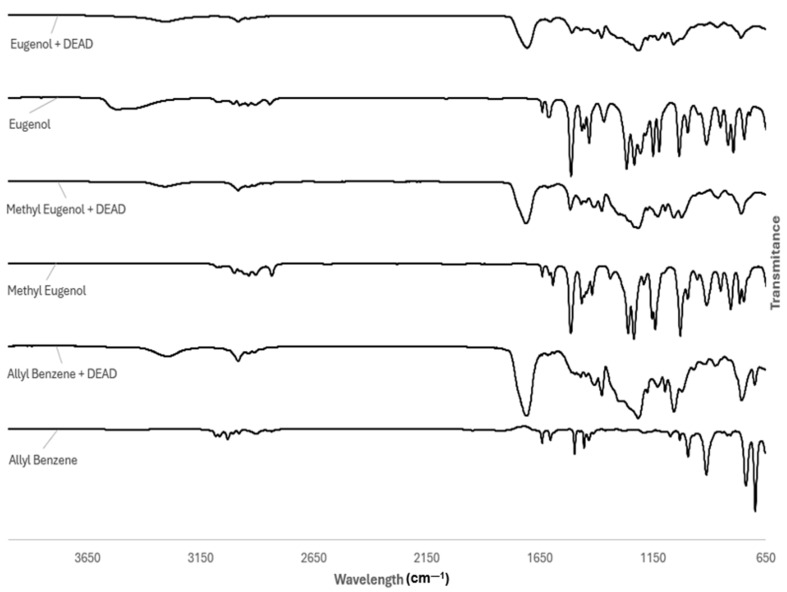
FTIR spectra of starting alkenes and ene reaction products (at 1:2 molar ratio of alkene/DEAD). From bottom to top: AB and AB-DEAD (sample A-2), ME and ME-DEAD (sample M-2), and eugenol and eugenol/DEAD (sample E-2).

Products A-2, M-2, and E-2 and the starting alkenes (allylbenzene, methyl eugenol, and eugenol) were analyzed using FTIR spectroscopy (Figure 6) to confirm the functional groups of the products in the analytical region and to characterize the fingerprint region of these new products. FTIR has been useful in characterizing allylbenzene derivatives with unusual chemistry [33,34]. The spectrum for A-2 shows that the product has a peak at 3250 cm^−1^, associated with N-H stretching of the NH-CO formed from the ene reaction of DEAD and allylbenzene. This peak is not found in the allylbenzene starting material. The narrow peak at 3000 cm^−1^ is associated with the C-H stretching of alkene groups. The intense peak at 1700 cm^−1^ is associated with C=O stretching and is characteristic of the DEAD moiety. The fingerprint region (400–1500 cm^−1^) exhibits many differences in peak intensity and wavelength compared to the starting material, inferring the product has several changes in vibrational modes compared to the reactant.

A similar trend is found in the FTIR spectrum of product M-2 compared to the starting material methyl eugenol. An N-H stretching peak is found at 3250 cm^−1^, which indicates the formation of the ME-DEAD ene adduct. Several peaks are found between 3000 and 2800 cm^−1^, which indicates C-H stretching in several types of alkene groups present in the product. The intense peak at 1700 cm^−1^ is associated with the carbonyl of the reacted ME-DEAD ene product. The fingerprint region indicates several changes in the structure compared to the ME starting material.

The spectrum for eugenol is somewhat similar to that of methyl eugenol; a major difference is the broad peak at ca. 3480 cm^−1^ in eugenol, corresponding to the O-H stretch, which is absent in methyl eugenol. The spectrum of product E-2 is similar to that of M-2, except that the spectrum for E-2 seems to have more rounded features in the fingerprint region, suggesting the presence of small overlapping peaks from other species. Overall, the FTIR spectra of the ene reaction products are consistent with the NMR analysis.

### 2.4. Physical Observations and Viscosity

**Figure 7:**
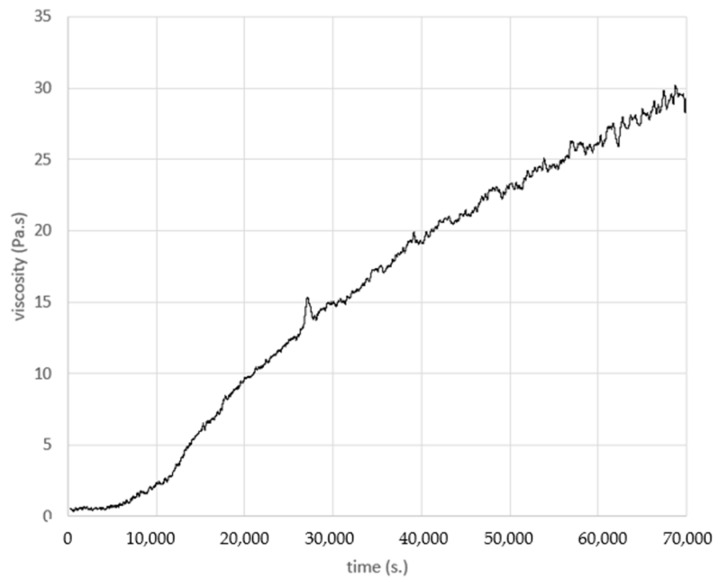
Viscosity buildup (in Pa-s) during the reaction of AB and DEAD as a function of reaction time (in seconds) at 87 °C for 19.4 h.

During the reactions of DEAD with the three phenylpropanoid compounds, the viscosities of the reaction mixtures were found to increase substantially. Initially, each mixture of the starting alkene and DEAD exhibited relatively low viscosity, but as the reaction progressed, the viscosity steadily rose to much higher levels. For example, viscosity as a function of time at 87 °C is shown in Figure 7 for the reaction of AB and DEAD at a 1:2 molar ratio. Over the course of 19.4 h, the viscosity of this reaction mixture increased from less than 1 Pa-s to about 28 Pa-s. The viscosity increase was enhanced with a longer reaction time or with a higher reaction temperature.

In order to study the viscosity phenomenon further, we prepared 11 samples with different weight levels of DEAD, as shown in Table 3. It is of interest that the weight ratio of DEAD used has a direct impact on the physical form of the final product. Thus, for the four samples in the ME series, the products range from a viscous liquid to a hard solid when an increasing amount of DEAD is used (Table 3, last column).

**Figure 8:**
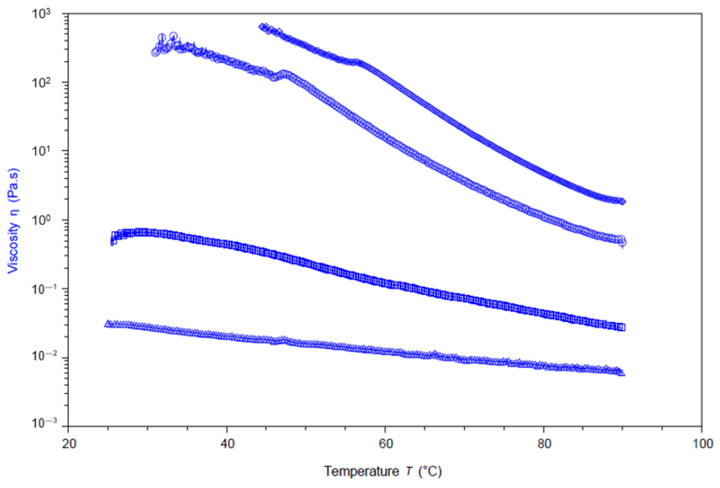
Viscosity of the AB-DEAD reaction products at different wt % DEAD measured as a function of increasing temperature; the samples are (from bottom to top) A-R1, A-R2, A-R3, and A-R4.

We took the 11 products shown in Table 3 and measured their viscosity as a function of increasing temperature. The results for the AB-DEAD products (A-R1, A-R2, A-R3, A-R4) are shown in Figure 8. Sample A-R1 is a liquid at 25 °C, and the viscosity is relatively low. Sample A-R2 is a viscous liquid with a higher viscosity than Sample A-R1. Sample A-R3 is a sticky solid, and no viscosity can be measured until melting at ca. 34 °C. Sample A-R4 is a hard sticky solid, and its viscosity is measured after melting at ca. 43 °C. In all cases, the viscosity decreases as the temperature increases, as expected. Furthermore, the products with higher levels of DEAD give greater hardness, higher viscosities, and greater reductions in viscosity upon heating. Thus, the ene reaction products seem to exhibit strong molecular aggregations, with higher DEAD levels enhancing these aggregations but higher temperatures reducing the aggregation.

**Figure 9:**
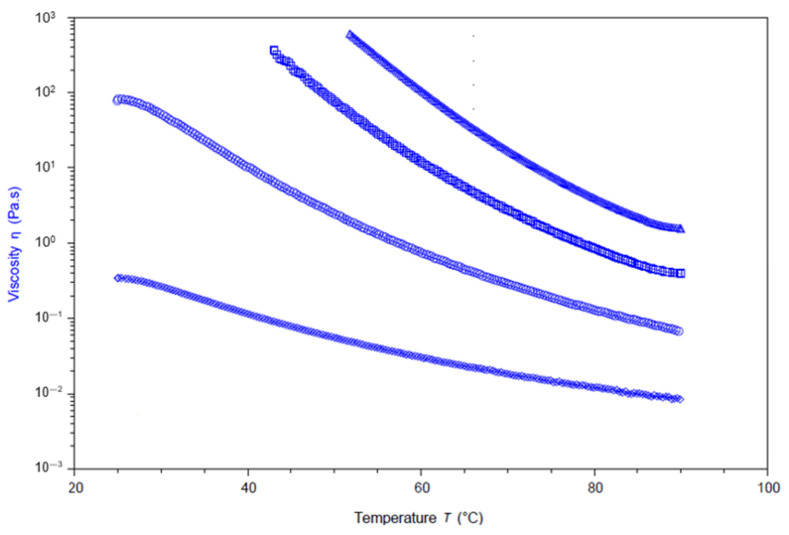
Viscosity of the ME-DEAD reaction products at different wt % DEAD measured as a function of temperature; the samples are (from bottom to top) M-R1, M-R2, M-R3, and M-R4.

**Figure 10:**
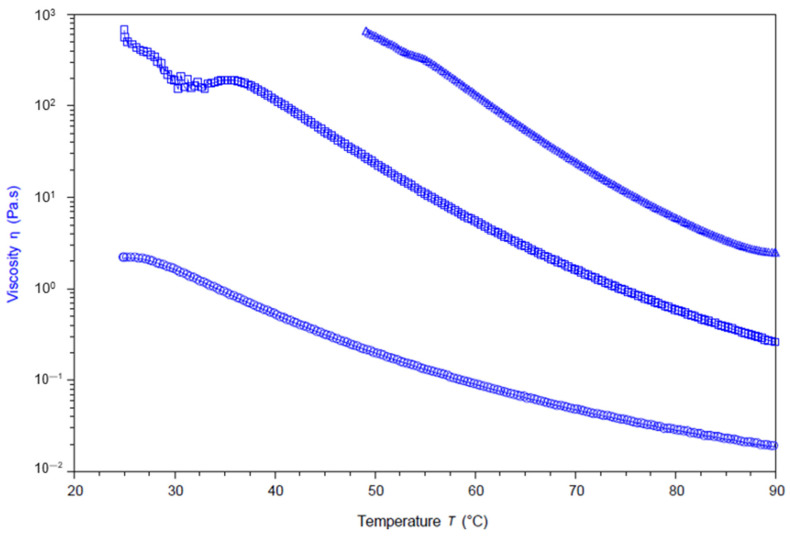
Viscosity of the eugenol/DEAD reaction products at different wt % DEAD measured as a function of temperature; the samples are (from bottom to top) E-R1, E-R2, and E-R3.

The viscosity versus temperature data for the ME-DEAD products (M-R1, M-R2, M-R3, M-R4) are shown in Figure 9. Sample M-R1 is a viscous liquid, with viscosity showing a steady reduction with increasing temperatures. Sample M-R2 is a soft solid, where the viscosity shows a slight plateau below 28 °C but decreases steadily thereafter. Samples M-R3 and M-R4 are both solids that melt at around 43 °C and 52 °C, respectively. For these samples, again the viscosity decreases as the temperature increases, and higher levels of DEAD in a product give greater hardness, higher viscosities, and greater negative slopes in the viscosity–temperature curves. Figure 10 shows the analogous curves for the eugenol/DEAD products (E-R1, E-R2, E-R3). Here again, the same trends as observed for the AB-DEAD and ME-DEAD products are found.

Although the above 11 products exhibited different physical forms at room temperature, they all dissolved in toluene. The addition of a small amount of toluene produced viscous solutions. However, further toluene additions resulted in major drops in viscosity. Table 4 shows the viscosity measured for the ene products at different toluene concentrations at 25 °C. All three ene products are solids at room temperature. At 25% dilution, the eugenol/DEAD product shows the highest viscosity at 3.7 Pa-s and the ME-DEAD product the lowest viscosity at 0.59 Pa-s. At 50% dilution, all samples show considerably reduced viscosity; for the eugenol/DEAD product, the viscosity at 50% dilution is less than 1% of the viscosity at 25% dilution, whereas for the other two products, 50% dilution reduces the viscosity to about 3% of the 25% dilution value. At 75% toluene, the viscosities are similar to that of water (1 mPa-s). At 90% dilution, the viscosities are similar to that of toluene (0.55 mPa-s) or slightly higher.

The exact nature of the increased viscosity exhibited by the ene products is still being investigated. One possibility is that the ene product molecules interact strongly with one another through aggregation, creating a three-dimensional network that leads to high viscosity. When diluted, these interactions are interspersed or disrupted by solvent molecules, thereby reducing viscosity. In our earlier studies involving the ene reactions between triglycerides and DEAD [5], triglycerides and PTAD [6], and cardanol and DEAD [7], increased viscosities were also observed for the ene reaction products. Possibly some of the same interactions are operative in all these cases.

### 2.5. Comments

There are many possible applications for a compound that exhibits high viscosity in its neat form but becomes significantly thinner when diluted with a solvent. For example, it may be used as a thickening agent in organic solvent formulations, such as paints, sealants, coatings, and adhesives. It may also be considered for post-application control, such that a solvent formulation containing this compound is applied to a surface, and when the solvent evaporates, the compound reverts to its more viscous form, providing a strong bond or durable coating.

In the literature, there are a large number of reactions that have been reported for eugenol for different applications [19,35,36]. Our current reaction with DEAD provides an additional, potentially important reaction to this collection. Moreover, the use of DEAD and PTAD as ene reagents for eugenol and other phenylpropanoid compounds in combination with those other reactions will likely produce further opportunities for new syntheses and additional applications.

## 3. Materials and Methods

### 3.1. Materials

Allylbenzene, eugenol, methyl eugenol, toluene, hexane and ethyl acetate were acquired from Sigma Aldrich (St Louis, MO, USA). DEAD was purchased from Fisher Scientific (Pittsburgh, PA, USA). For the NMR analysis, deuterochloroform was obtained from Cambridge Isotope Laboratories (Andover, MA, USA).

### 3.2. Preparative Procedures

For the reactions involving allylbenzene/DEAD, methyl eugenol/DEAD, and eugenol/DEAD, the two starting reagents in a fixed molar ratio were placed with stirring into a 10 mL Reacti-Vial, which was then heated for 6–22 h in a Pierce Reacti-Therm stirrer/heater module at 80–100 °C. The mixture became thick upon extended heating, and stirring could be difficult. At the end of the reaction, the product was removed from the vial and analyzed without further purification.

Selected products from the syntheses were purified using a silica gel column with a hexane/ethyl acetate (1:3 *v*/*v*) eluent. The column had a diameter of 2.5 cm and was packed with silica gel to a length of 25 cm. In a typical purification procedure, the crude product (150 mg) was loaded at the top of the silica gel in the column. The eluent was passed through the column at a rate of 5 mL/min. The progress of the purification was monitored using thin layer chromatography (TLC) with the same solvent system, hexane/ethyl acetate (1:3 *v*/*v*). The solvent containing the desired product was evaporated and dried under vacuum to yield purified products.

### 3.3. Nuclear Magnetic Resonance Spectroscopy (NMR)

The ^1^H and ^13^C NMR solution spectra were collected with a Bruker Avance 500 spectrometer (500.11 MHz for ^1^H) using a 5-mm BBO probe. All samples were dissolved in CDCl_3_. Chemical shifts were reported as ppm from tetramethylsilane as the reference. Standard operating procedures were used, with the probe set at 27 °C and the spectra processed with the Bruker Topspin v1.3 software. The COSY and the HSQC spectra were obtained using the Bruker standard pulse sequences.

### 3.4. Fourier Transform Infrared Spectroscopy (FTIR)

The FTIR data were obtained on a PerkinElmer spectrometer (Waltham, MA, USA) using a universal ATR sampling accessory. Sixteen scans were obtained by scanning from 650–4000 cm^−1^, with a 4 cm^−1^ resolution and at room temperature. The collected data were transferred from the instrument to a computer and processed with an Excel spreadsheet (Microsoft, Redmond, WA, USA).

### 3.5. Viscosity Measurements

The instrument used was an ARES G2 rheometer from TA Instrument (New Castle, DE, USA). For the viscosity versus time study, the alkene/DEAD mixture was placed in a 25 mm parallel plate geometry at 87 °C, with the plate operated at an angular oscillatory frequency of 6.28 rad per second. The viscosity of the reaction mixture was measured for about 20 h. As for the viscosity versus temperature curves of the products, each product was placed in a 25 mm parallel plate with a gap of 1.4 mm. The temperature was then ramped up from 25 °C to 90 °C while the viscosity was measured. For the viscosity dilution study, the ene reaction products made at the 1:2 alkene/DEAD molar ratio were dissolved using different amounts of toluene. Each toluene solution was placed in a 25 mm parallel plate geometry at 25 °C, with the plate operated at a constant shear rate of 100 s^−1^. Viscosity values collected at the first 15 s were averaged and reported.

### 3.6. Elemental Analyses

Elemental analyses were conducted at Galbraith Laboratories, Inc., Knoxville, TN, USA. The instrument for the carbon, hydrogen, and nitrogen analyses was a PerkinElmer 2400 Series II CHNS/O Analyzer. This instrument burned the samples in pure oxygen at 920–980 °C under static conditions to produce combustion products of CO_2_, H_2_O, and N_2_. The PE-2400 automatically separated and analyzed these products in a self-integrating, steady-state thermal conductivity analyzer. For oxygen analysis, a Thermo Finnigan FlashEA 1112 Elemental Analyzer was used. The sample material was pyrolyzed under an inert atmosphere and passed over a nickelized carbon catalyst, converting organic oxygen species to carbon monoxide. The carbon monoxide was then separated by a chromatographic column, analyzed by a thermal conductivity detector, and then calculated as oxygen content based on external calibration.

## 4. Conclusions

Our motivation for this work stems from a strong interest in utilizing agro-based materials as green and sustainable resources, particularly through the ene reaction, which offers the advantage of being a one-step process without the need for a catalyst or the formation of by-products. Given the diverse pharmacological and industrial applications of phenylpropanoids, we aimed to explore further opportunities for their use through derivatization reactions. Notably, all three phenylpropanoids (allylbenzene, methyl eugenol, and eugenol) readily undergo the predominant ene reaction. For allylbenzene and methyl eugenol, the ene derivatives are the primary products up to the alkene-to-DEAD molar ratio of approximately 1:2. Eugenol also reacts with DEAD to form an ene product, but there are additional products formed. Interestingly, these ene derivatives exhibit significant increases in viscosity, which is believed to be due to strong intermolecular interactions in the ene product. However, when toluene (as a solvent) is added to the product, the viscosity decreases significantly. This self-aggregation property may enable these products to be useful in thickening and viscosity control applications for industrial formulations containing organic solvents.

## Figures and Tables

**Table 1:** Selected products made with DEAD and allylbenzene (AB), methyl eugenol (ME), and eugenol.

Sample Code	Alkene	Wt. of Alkene (mg)	Wt. of DEAD (mg)	Molar Ratio Alkane:DEAD
A-1	AB	100	160	1:1.09
A-2	AB	100	320	1:2.17
A-3	AB	100	360	1:2.44
A-4	AB	100	440	1:2.98
A-5	AB	100	480	1:3.25
A-6	AB	100	640	1:4.34
M-1	ME	100	112	1:1.15
M-2	ME	100	225	1:2.30
E-1	eugenol	100	106	1:1
E-2	eugenol	100	212	1:2

**Table 2:** ^13^ C chemical shifts (ppm) of allylbenzene (U) and its DEAD product (A); methyl eugenol (V) and its DEAD product (G), and eugenol (I) and its DEAD product (H); peak numbering is shown in the structures in Scheme 3, Scheme 4 and Scheme 5.

Peak No.	U	A	V	G	I	H (est’d)
1	140.2	136.6	132.6	129.6	132.0	126.8
2	128.8	126.1	120.5	119.7	114.4	113.0
3	128.6	128.3	147.4	149.0	146.6	149.7
4	126.3	127.9	148.9	149.1	144.0	144.2
5			111.4	108.9	115.5	110.5
6			111.9	111.1	121.3	119.7
7	40.5	133.6	39.8	133.5	39.9	133.5
8	137.7	123.8	137.7	121.6	137.9	120.1
9	116.0	52.6	115.6	52.4	111.2	53
10		156.4		160.0		157.0
11		63.8		62.4		63.6
12		14.1		14.4		14.3

**Table 3:** Physical observations at room temperature of the products from the reaction (overnight at 90 °C) between three alkenes and DEAD at different DEAD levels.

Sample Code	Alkene	Alkene wt. Ratio	DEAD wt. Ratio	Alkene:DEAD Molar Ratio	Observation at Room Temperature
A-R1	AB	100	48	1:0.33	Liquid
A-R2	AB	100	97	1:0.66	Viscous liquid
A-R3	AB	100	147	1:1.00	Sticky solid
A-R4	AB	100	196	1:1.33	Hard sticky solid
M-R1	ME	100	48	1:0.49	Viscous liquid
M-R2	ME	100	97	1:0.99	Soft solid
M-R3	ME	100	147	1:1.50	Hard sticky solid
M-R4	ME	100	196	1:2.01	Hard solid
E-R1	Eugenol	100	48	1:0.45	Viscous liquid
E-R2	Eugenol	100	97	1:0.92	Sticky solid
E-R3	Eugenol	100	147	1:1.39	Hard solid

**Table 4:** Viscosity of diluted ene products from the DEAD reaction (at 1:2 molar ratios of alkene/DEAD). All viscosity measured at 25 °C.

Product wt. %	Toluene wt. %	Viscosity (mPa-s)		
		AB-DEAD	ME-DEAD	Eugenol/DEAD
100	0	(solid)	(solid)	(solid)
75	25	1004.8	590.7	3702.3
50	50	35.0	15.8	31.0
25	75	1.1	1.1	1.4
10	90	0.6	0.5	1.3

## Data Availability

Data supporting the findings are available upon reasonable request to the corresponding authors.

## Supplementary Materials

The following supporting information can be downloaded at: https://www.mdpi.com/article/10.3390/molecules30050977/s1. Figure S1. NMR spectra of the ene reaction products between allylbenzene and DEAD (sample A-2): (a) ^1^H spectrum with integration; (b) 2D COSY plot (^1^H-^1^H shift correlation); (c) 2D HSQC (^1^H-^13^C shift correlation). Figure S2. NMR spectra of the purified ene reaction product between allylbenzene and DEAD: (a) ^13^C spectrum (upper plot), (b) ^1^H spectrum (lower plot). The letter A denotes the ene product, and the subscripts correspond to the numbering shown in Scheme 3. The letter X indicates the CDCl_3_ peaks. Figure S3. NMR spectra of the ene reaction products between methyl eugenol and DEAD (sample M-1): (a) ^1^H spectrum with integration; (b) 2D COSY plot (^1^H-^1^H shift correlation); (c) 2D HSQC (^1^H-^13^C shift correlation). Figure S4. ^1^H NMR spectrum (with integration) of the reaction products between eugenol and DEAD (sample E-1).
