# Supplementary material for: Self-Thickening Materials Derived from Phenylpropanoid Ene Reactions
Source: Molecules. 2025 Feb 20;30(5):977. doi: 10.3390/molecules30050977 (PMC11901909; doi:10.3390/molecules30050977)
Supplement: Supplementary file 1 [file molecules-30-00977-s001.zip › molecules-3257820-supplementary.pdf]

# Self-Thickening Materials derived from Phenylpropanoid Ene Reactions

**Atanu Biswas [1,*], H. N. Cheng [2,*], Bret Chisholm [1], Ryan Beni [1,3], Zengshe Liu [1], Karl Vermillion [1], Michael Appell [1], Kelton Forson [1], Omar El Seoud [4], Carlucio R. Alves [5], Roselayne F. Furtado [6]**

[1] National Center for Agricultural Utilization Research, USDA Agricultural Research Services, 1815 N. University Street, Peoria, Illinois 61604, United States
[2] Southern Regional Research Center, USDA Agricultural Research Service, 1100 Allen Toussaint Boulevard, New Orleans, Louisiana 70124, United States
[3] Department of Chemistry, Tennessee State University, Nashville, Tennessee 37209, USA
[4] Institute of Chemistry, University of São Paulo, 05508-000 São Paulo, SP, Brazil
[5] State University of Ceará, Chemistry Department, Silas Munguba Av. 1.700, 60740-020, Fortaleza, CE, Brazil
[6] Embrapa Agroindústria Tropical, Rua Dra. Sara Mesquita 2270, CEP 60511-110 Fortaleza, CE, Brazil

* Correspondence: atanu.biswas@usda.gov (A.B.); hncheng100@gmail.com (H.N.C.)

## Supplementary Materials

Figure S1. NMR spectra of the ene reaction products between allylbenzene and DEAD (sample A-2): a) $^{1}$H spectrum with integration; b) 2D COSY plot ($^{1}$H-$^{1}$H shift correlation); c) 2D HSQC ($^{1}$H-$^{13}$C shift correlation).

Figure S2. NMR spectra of the purified ene reaction product between allylbenzene and DEAD: a) $^{13}$C spectrum (upper plot), b) $^{1}$H spectrum (lower plot). The letter A denotes the ene product and the subscripts correspond to the numbering shown in Scheme 3. The letter X indicates the $CDCl_3$ peaks.

Figure S3. NMR spectra of the ene reaction products between methyl eugenol and DEAD (sample M-1): a) $^{1}$H spectrum with integration; b) 2D COSY plot ($^{1}$H-$^{1}$H shift correlation); c) 2D HSQC ($^{1}$H-$^{13}$C shift correlation).

Figure S4. $^{1}$H NMR spectrum (with integration) of the reaction products between eugenol and DEAD (sample E-1).

S1a

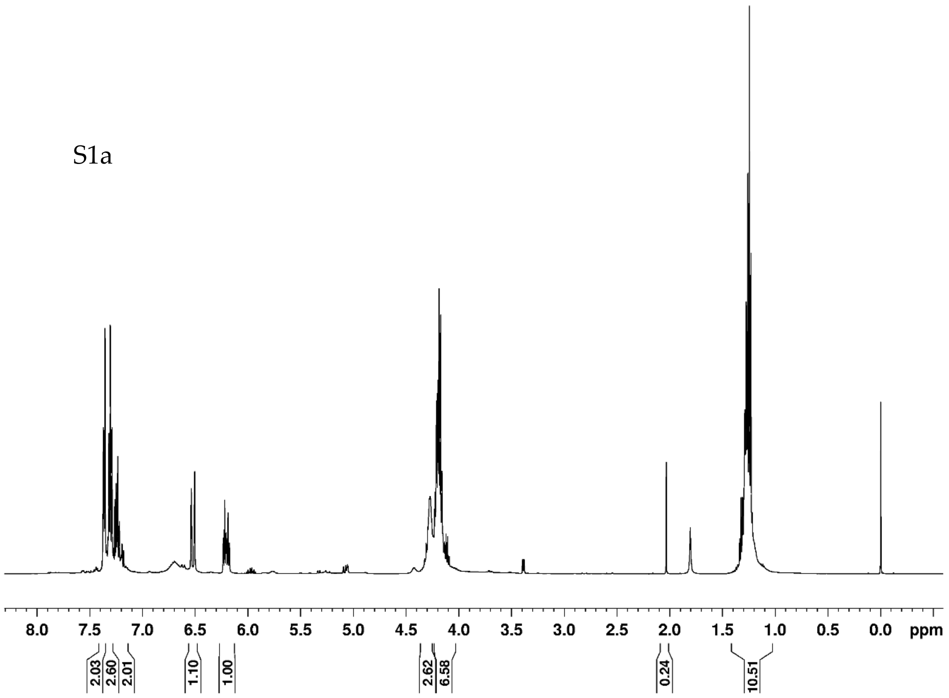
8.0 7.5 7.0 6.5 6.0 5.5 5.0 4.5 4.0 3.5 3.0 2.5 2.0 1.5 1.0 0.5 0.0 ppm
2.03 2.60 2.01 1.10 1.00 2.62 6.58 0.24 10.51

S1b

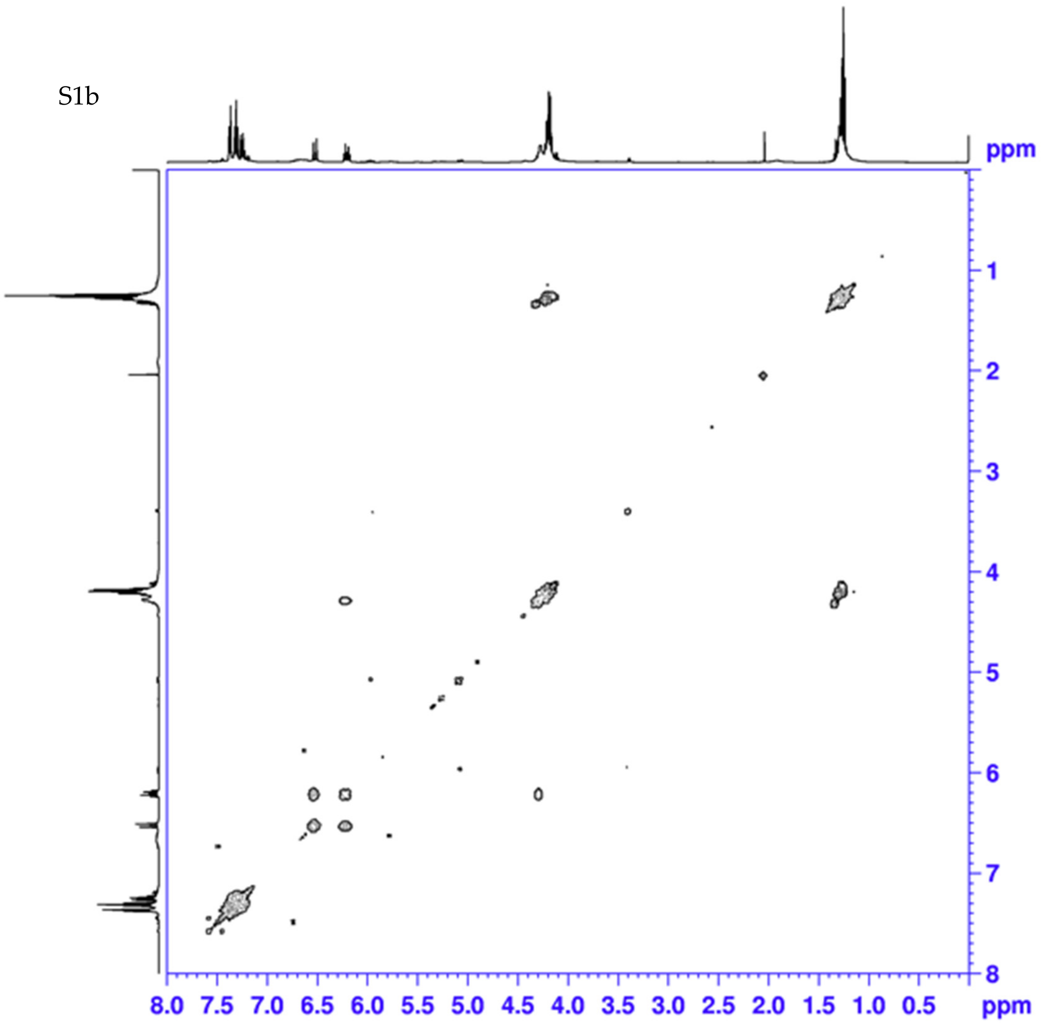
ppm
1 2 3 4 5 6 7 8
8.0 7.5 7.0 6.5 6.0 5.5 5.0 4.5 4.0 3.5 3.0 2.5 2.0 1.5 1.0 0.5 ppm

S1c

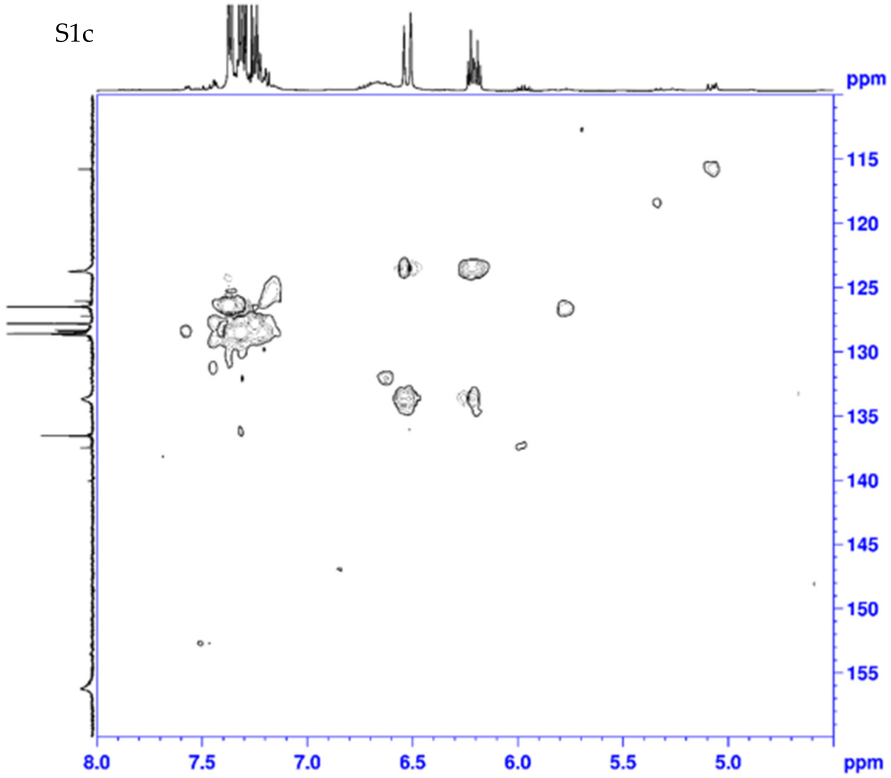


Figure S1. NMR spectra of the ene reaction products between allylbenzene and DEAD (sample A-2): a) $^{1}H$ spectrum with integration; b) 2D COSY plot ($^{1}H$-$^{1}H$ shift correlation); c) 2D HSQC ($^{1}H$-$^{13}C$ shift correlation).

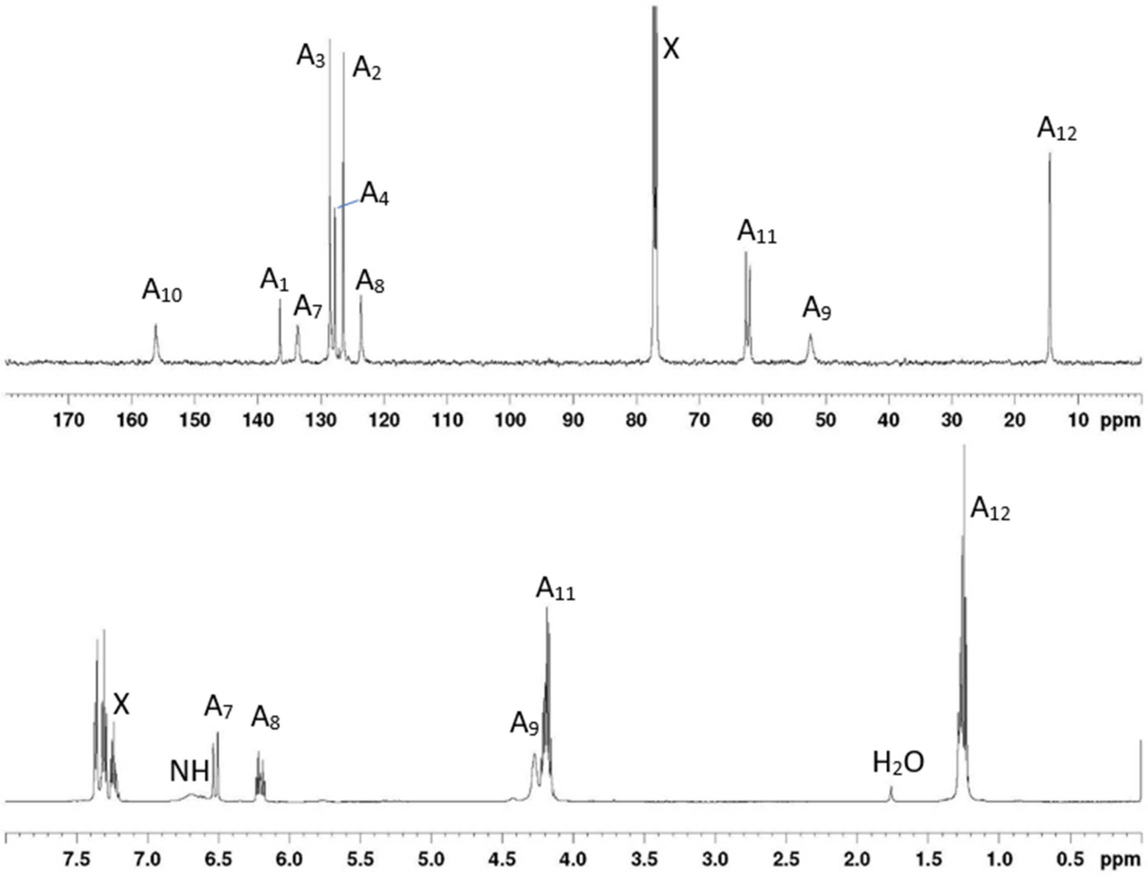


Figure S2. NMR spectra of the purified ene reaction product between allylbenzene and DEAD: a) $^{13}C$ spectrum (upper plot), b) $^{1}H$ spectrum (lower plot). The letter A denotes the ene product and the subscripts correspond to the numbering shown in Scheme 3. The letter X indicates the $CDCl_3$ peaks.

S3a

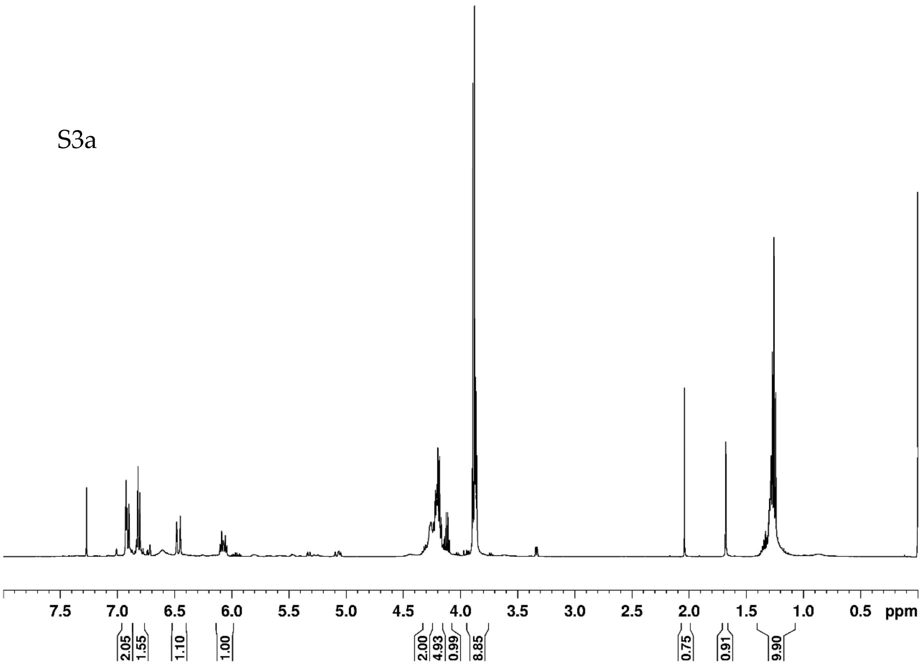
7.5 7.0 6.5 6.0 5.5 5.0 4.5 4.0 3.5 3.0 2.5 2.0 1.5 1.0 0.5 ppm
2.05
1.55
1.10
1.00
2.00
4.93
0.99
8.85
0.75
0.91
9.90

S3b

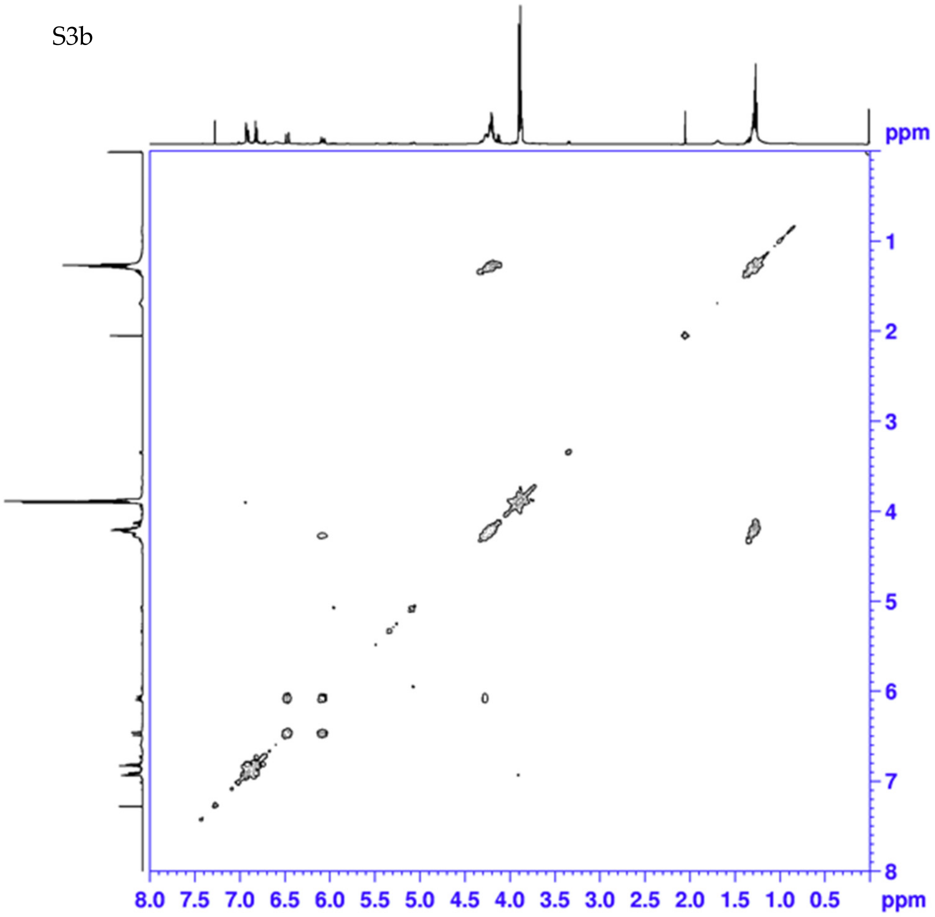
ppm
1
2
3
4
5
6
7
8
8.0 7.5 7.0 6.5 6.0 5.5 5.0 4.5 4.0 3.5 3.0 2.5 2.0 1.5 1.0 0.5 ppm

S3c

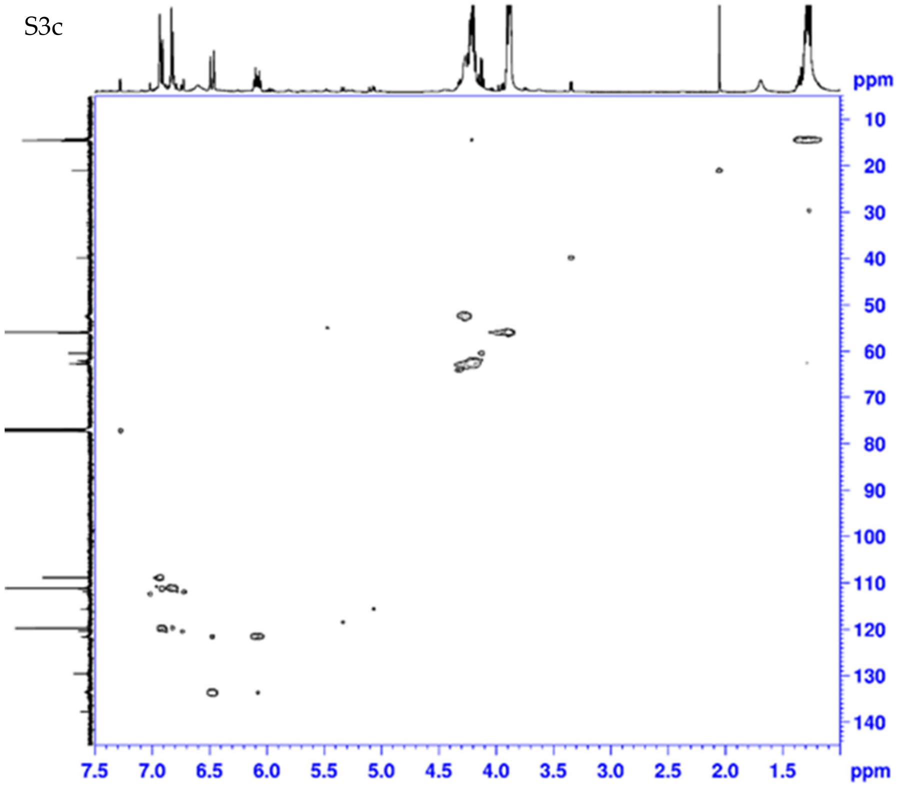


Figure S3. NMR spectra of the ene reaction products between methyl eugenol and DEAD (sample M-1): a) $^{1}H$ spectrum with integration; b) 2D COSY plot ($^{1}H$-$^{1}H$ shift correlation); c) 2D HSQC ($^{1}H$-$^{13}C$ shift correlation).

S4

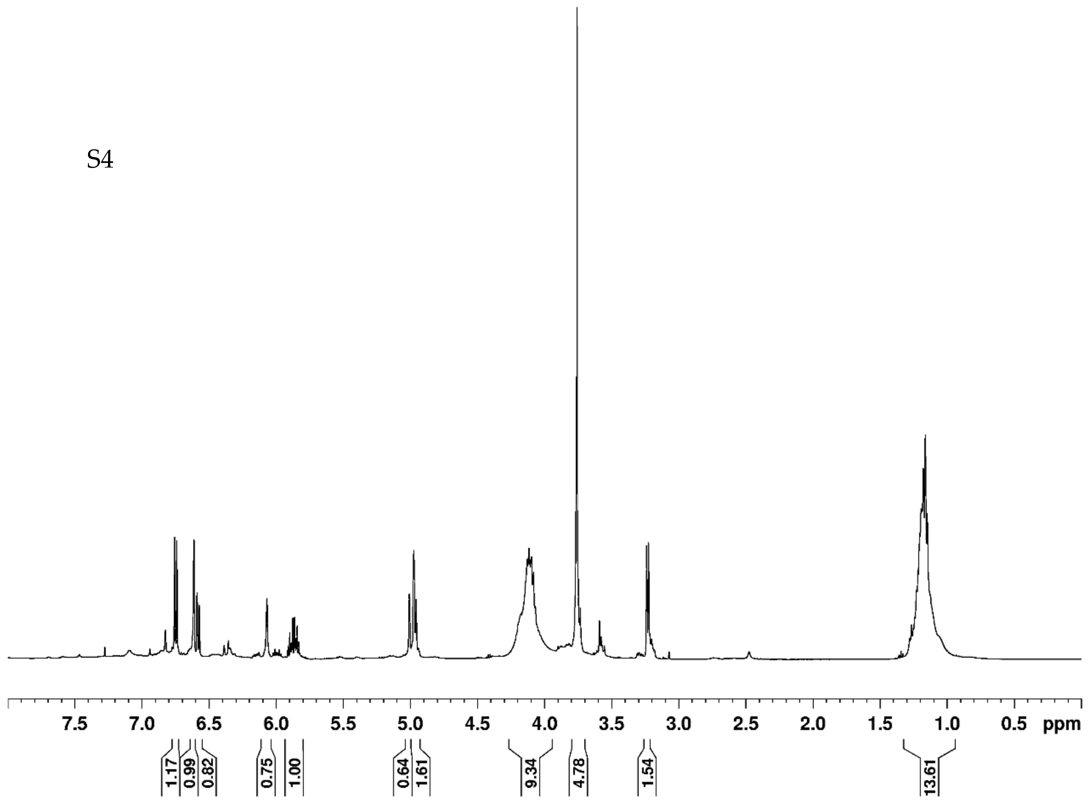


Figure S4. $^{1}$H NMR spectrum (with integration) of the ene reaction products between eugenol and DEAD (sample E-1).
